# Selective gene expression profiling contributes to a better understanding of the molecular pathways underlying the histological changes observed after RHMVL

**DOI:** 10.1186/s12920-022-01364-z

**Published:** 2022-10-07

**Authors:** Janine Arlt, Sebastian Vlaic, Ronny Feuer, Maria Thomas, Utz Settmacher, Uta Dahmen, Olaf Dirsch

**Affiliations:** 1 Experimental Transplantation Surgery, Department of General, Visceral and Vascular Surgery, Jena University Hospital, Drackendorfer Str. 1, 07747 Jena, Germany; 2 Leibniz Institute for Natural Product Research and Infection Biology Hans Knöll Institute (HKI), Beutenbergstraße 11a, 07745 Jena, Germany; 3 Institute for System Dynamics, University of Stuttgart, Pfaffenwaldring 9, 70569 Stuttgart, Germany; 4 Dr. Magarete Fischer-Bosch Institute for Clinical Pharmacology, Auerbachstr. 112, 70376 Stuttgart, Germany; 5 Department of General, Visceral and Vascular Surgery, Jena University Hospital, Erlanger Allee 101, 07747 Jena, Germany; 6 Institute of Pathology, Jena University Hospital, Ziegelmühlenweg 1, 07743 Jena, Germany

**Keywords:** Liver resection, Portal hypertension, Gene expression, Vasoactive drug

## Abstract

**Background:**

In previous studies, five vasoactive drugs were investigated for their effect on the recovery process after extended liver resection without observing relevant improvements. We hypothesized that an analysis of gene expression could help to identify potentially druggable pathways and could support the selection of promising drug candidates.

**Methods:**

Liver samples obtained from rats after combined 70% partial hepatectomy and right median hepatic vein ligation (*n* = 6/group) sacrificed at 0 h, 24 h, 48 h, and 7days were selected for this study. Liver samples were collected from differentially perfused regions of the median lobe (obstruction-zone, border-zone, normal-zone). Gene expression profiling of marker genes regulating hepatic hemodynamics, vascular remodeling, and liver regeneration was performed with microfluidic chips. We used 3 technical replicates from each sample. Raw data were normalized using LEMming and differentially expressed genes were identified using LIMMA.

**Results:**

The strongest differences were found in obstruction-zone at 24 h and 48 h postoperatively compared to all other groups. mRNA expression of marker genes from hepatic hemodynamics pathways (iNOS,Ptgs2,Edn1) was most upregulated.

**Conclusion:**

These upregulated genes suggest a strong vasoconstrictive effect promoting arterial hypoperfusion in the obstruction-zone. Reducing iNOS expression using selective iNOS inhibitors seems to be a promising approach to promote vasodilation and liver regeneration.

**Supplementary Information:**

The online version contains supplementary material available at 10.1186/s12920-022-01364-z.

## Introduction

Extended liver resection leads to a reduction of functional liver mass beyond the surgical loss. Extended liver resection leads to portal hypertension and arterial hypoperfusion of the liver remnant [1, 2]. Furthermore, extended liver resection requires transection of the middle hepatic vein and leads to focal outflow obstruction. Together, arterial hypoperfusion and focal outflow obstruction compromise the perfusion of the liver remnant. This leads to pericentral necrosis of the undrained territories and subsequently to an additional loss of functional liver mass [3–5]. The additional loss of functional liver mass after extended liver resection may lead to hepatic insufficiency and ultimately cause the death of the patient [6–9].

Portal hypertension after extended liver resection can be treated by operative interventions in clinical practice. Additional procedures such as splenectomy are performed to reduce the surgically induced portal hypertension. However, performing an additional procedure may increase the overall risk for complications [10–14].

Portal hypertension, especially in chronic cirrhotic liver disease, can be treated by drug administration. Drugs acting as vasodilators such as non-selective b-blockers (propranolol, carvedilol), nitric oxide (NO) donors (isosorbide dinitrate, isosorbide-5-mononitrate), or drugs to decrease portal venous blood flow (terlipressin, octreotide) are administered for the treatment of portal hypertension [15–18]. Pharmacological treatment of portal hypertension after extended liver resection may prevent the complications induced by additional operative interventions and should be further investigated.

Liver regeneration after extended liver resection is a complex process. The complexity is due to the simultaneous activation of interwoven signaling mechanism. On one hand, liver resection leads to the activation of liver regeneration to restore the functional liver mass. On the other hand, the imbalance of hepatic hemodynamics leads to the activation of vasoactive mechanisms and vascular remodeling to restore hepatic venous drainage [4, 19–25].

The interwoven signaling mechanisms can be grouped as follows:

- *Hepatic hemodynamics* Regulation of hepatic hemodynamics is controlled by different vasoactive mechanisms. Vasoactive mechanisms are activated by portal hypertension and arterial hypoperfusion. The main mechanisms are the adenosine-based “Hepatic arterial buffer response” (HABR), NO pathway, the endothelin pathway, and the arachidonic acid pathway [20, 22, 23].
- *Vascular remodeling* Focal outflow obstruction induces a vascular remodeling process. The liver restores hepatic venous drainage through the formation of sinusoidal vascular canals in the border zone (BZ) between the outflow obstruction zone (OZ) and the normal zone (NZ) [3, 24, 26].
- *Liver regeneration* Liver regeneration is induced in both, the drained NZ and the OZ, by the loss of liver mass and the resulting portal hyperperfusion and parenchymal necrosis. The essential factors involved in liver regeneration are encompassed by three types: cell cycle-associated marker genes, hepatocyte growth factors, and cytokines. The signaling mechanisms of liver regeneration have been described in detail, e.g., by Fausto et al. (2006), Michalopoulos (2007), and Riddiough et al. (2021) [19, 21, 25].

First insights into the spontaneous recovery process of outflow obstruction after extended liver resection were previously obtained in a newly developed surgical model in rats combining focal hepatic outflow obstruction (FHOO) and extended liver resection. Dirsch et al. (2008) and Huang et al. (2014) used this model to investigate liver recovery after extended liver resection. They induced FHOO by right median hepatic vein ligation (RMHVL) performed in the same operation as the partial hepatectomy (PHx). They showed that revascularization via the formation of sinusoidal canals as well as liver mass restoration was completed within a week after the surgical intervention. During the postoperative recovery and regeneration phase, confluent pericentral necrosis was resorbed and replaced by proliferating hepatocytes [3, 26].

Huang et al. (2011) investigated the role of arterial blood supply by comparing the spontaneous recovery from hepatic outflow obstruction in a non-arterialized versus arterialized partial liver transplantation model. He demonstrated that the lack of hepatic arterial perfusion determined the extent of hepatic necrosis in OZ [4].

Furthermore, Huang et al. (2014) investigated the effect of modulating hepatic arterial perfusion by influencing the NO pathway in the surgical model of extended liver resection with FHOO [26]. The molecular compound NO increases the hepatic arterial flow by arterial vasodilation [26–28]. However, the application of molsidomine, a NO donor, did not show the expected beneficial effect. In contrast, the application of N(*ω*)-nitro-L-arginine methyl ester (L-NAME), a competitive NO synthase (NOS) inhibitor, reduced hepatic arterial flow, increased parenchymal necrosis, and delayed spontaneous recovery, as expected [26].

In a subsequent study, five clinically established vasoactive drugs were investigated for their ability to improve hepatic perfusion in this situation. The selection was guided by a thorough knowledge-based literature work-up of more than 20 drug classes addressing different molecular pathways such as NO pathway, arachidonic acid pathway, and endothelin pathway. However, despite a modulatory effect on hepatic hemodynamics, no relevant improvement of hepatic damage was observed [9, 29].

We hypothesized that an analysis of gene expression could help to identify potentially druggable pathways and could support the selection of promising drug candidates. Therefore, this study aimed to identify regulated signaling pathways based on selected marker genes.

Due to the numerous signal transduction pathways involved in the complex recovery process, only a selection of marker genes representing the different mechanisms was analyzed using quantitative high-throughput reverse transcription PCR (RT-qPCR). The selected genes are involved in regulatory processes underlying the regulation of hepatic hemodynamics, vascular remodeling, and liver regeneration. The simultaneous investigation of the respective gene expression levels allows for a hypothesis-driven analysis of signaling pathways in contrast to a transcriptome analysis.

We compared gene expression in healthy control animals and animals subjected to extended liver resection with RMHVL at different time points and in the three different zones. The gene expression analysis revealed highly regulated significantly differentially expressed potential marker genes and thereby the most affected pathways.

## Materials and methods

### Sample generation

For this gene expression study, we used a set of previously generated liver samples [26]. Male inbred Lewis (Lewis/HanTMHsd) rats (250–350 g, Central Animal Laboratory, University Hospital Essen, Germany) had been subjected to RMHVL in combination with 70% PHx. The left lateral lobe, the left median lobe, the superior plus inferior caudate lobes, and the right superior plus inferior lobes were resected, leading to an estimated 70% reduction of liver mass. Liver tissue samples were taken from the obstructed territory of the RMHV (OZ), normal zone (NZ), and border zone between OZ and NZ (BZ) and were snap-frozen immediately after surgery and stored in liquid nitrogen until used. Samples from 6 animals were taken at each observation time (0 h, 24 h, 48 h, 7days).

All procedures, experiments, and housing of the animals were carried out according to current German regulations and guidelines for animal welfare and to international principles of laboratory animal care, following the ARRIVE Guidelines Checklist as well. Ethics committee of Thüringer Landesamt für Verbraucherschutz, Thuringia, Germany, approved this animal study. The protocols were approved by the Thüringer Landesamt für Verbraucherschutz, Thuringia, Germany (Approval-Number: 02–023/14).

### Selection of marker genes for quantitative high-throughput RT-qPCR analysis

*First,* we performed a thorough literature review to identify the relevant biological mechanisms involved in regulatory processes underlying the regulation of hepatic hemodynamics, vascular remodeling, and liver regeneration.

*Second*, we selected 3–7 genes for each pathway, which resulted in a total of 37 marker genes. The following marker genes from the key signal transduction pathways (Table 1) were selected for the analysis:

**Table 1:** List of selected marker genes*

Mechanisms	Pathways	Genes	References (e.g.)
Vasoactive pathways	NO pathway	eNOS, iNOS, nNOS, Gucy1a2	[22], [28], [35]
	Arachidonic acid pathway	PLA2G4A, COX1, COX2, PGIS, TXS, Tbxa2r	[27], [36]
	Endothelin pathway	Edn1, ET-R_A,_ ET-R_B_	[27], [36], [37]
	Hepatic arterial buffer response”	Adora1, Adora2a, Adora3	[20], [23]
Vascular remodeling		CAV1, Icam1, Pecam1 Prdx1, vWF, Lamc2, Vegfa, Vegfb	[24], [37–39]
Liver regeneration	Liver cell proliferation	PCNA, Tyms	[40], [41]
	Liver cell growth factors	Egf, Egfr, Egr1, Hgf, Met	[19], [21, 25]
	Cytokines	IL10, IL1b, IL6, Mif, Tgfb1, Tnf	[22], [25], [36], [37]

^*^List of selected marker genes for each mechanism and pathway based on selected references

**Fig. 1:**
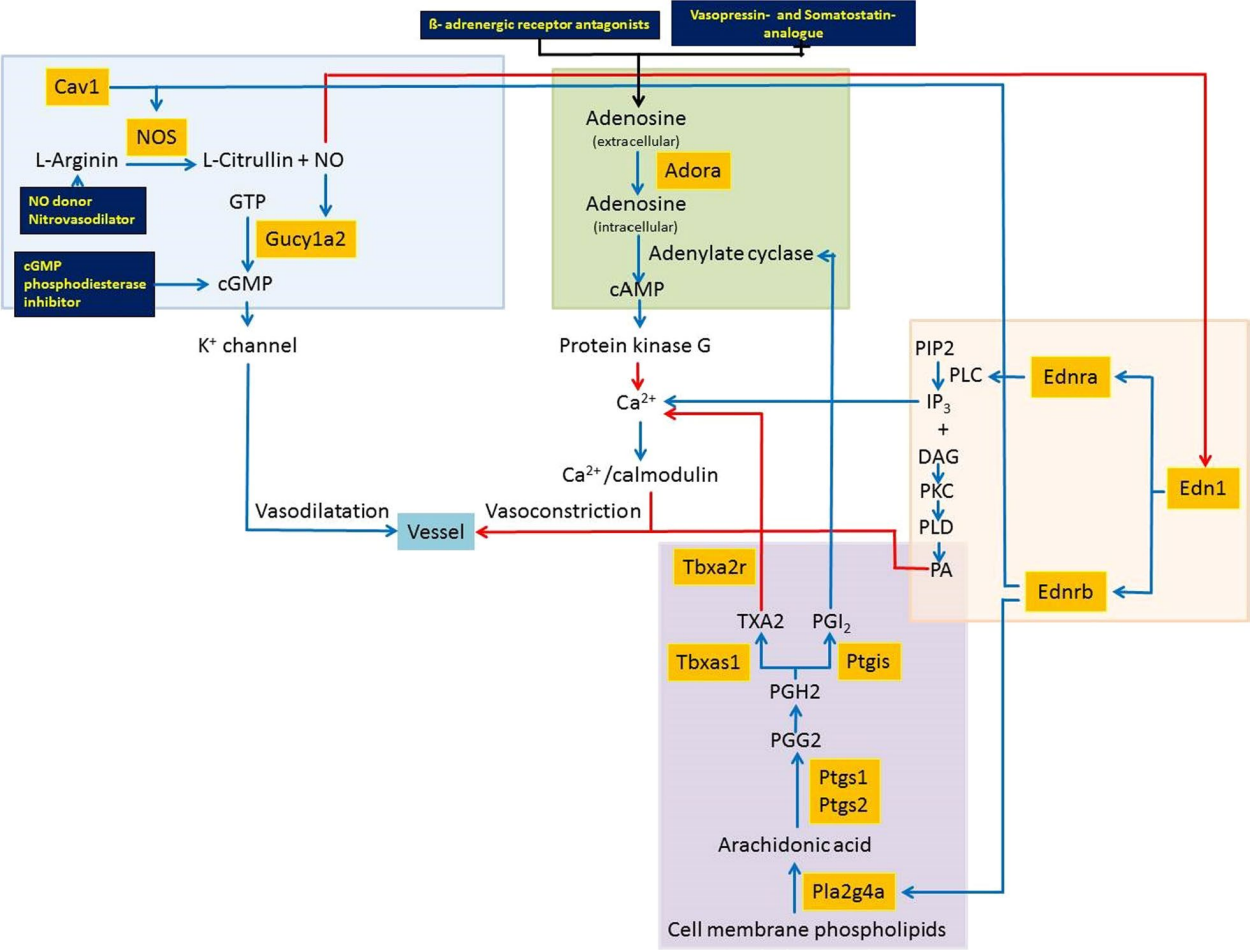
Diagram of different vasoactive molecular pathways involved in the regulation of hepatic hemodynamics. List of interactions between pathways: blue arrows—enhancing effect, red arrows—inhibitory effect; dark blue tiles: interrelated drug classes; yellow text—drug classes with substance investigated in previous studies; orange box—investigated genes, By courtesy of Springer Nature: Modified figure of previously published figure in Arlt et al. (2017) [29]. List of abbreviations: Adora, Adenosine receptor; Ca^2+^- Calcium; cAMP, cyclic adenosine monophosphate; cGMP, cyclic guanosine monophosphate; DAG Diacylglycerol; Edn1-Endothelin 1; Ednra, Endothelin receptor type A; nEdra, Endothelin receptor type B 1/2; GTP, Guanosine triphosphate; GucY1a2- Guanylate cyclase soluble subunit alpha-2; IP_3_, Inositol 1,4,5-trisphosphate; K^+^ -Potassium; NO, Nitric oxide; NOS, Nitric oxide synthases; PA- Phosphatidic acid; PGH2, Prostaglandin H2; PGI_2_, Prostacyclin; Ptgis -Prostacyclin synthase; PIP2, Phosphatidylinositol 4,5-bisphosphate; PKC- Protein kinase C; PLC -Phospholipase C; PLD, Phospholipase D; Ptgs 1/2 -Cyclooxygenase-1/2; TXA2- thromboxane A2; Tbxa2r -Thromboxane A2 Receptor; Tbxas1- Thromboxane synthase

For regulation of hepatic hemodynamics, we selected 16 marker genes from four vasoactive mechanisms. The selected marker genes of these four mechanisms were as follows: The NO pathway was represented by the marker genes eNOS, iNOS, nNOS, and Gucy1a2, the arachidonic acid pathway by the marker genes Pla2g4a, Ptgs1, Ptgs2, Ptgis, Tbxas1, Tbxa2r, the endothelin pathway by the marker genes Edn1, ET-R_A_, and ET-R_B_, and the adenosine-based HABR was represented by the marker genes Adora1, Adora2a, and Adora3 (Fig. 1).

For vascular remodeling, we considered the following 8 genes: CAV1, Vegfa, Vegfb, Icam1, Pecam1, Prdx1, vWF, and Lamc2 as marker genes.

For liver regeneration, we selected 13 marker genes including cell cycle-associated genes, hepatocyte growth factors, and cytokines. Cell cycle-associated marker genes were represented by PCNA and Tyms. The following growth factors and receptors were included: Egf, Egfr, Egr1, Hgf, and Met. We selected six cytokines (IL10, IL1b, IL6, Mif, TGFb1, and Tnf) relevant for liver regeneration.

The remaining 59 slots were used for other genes which were investigated in other studies in our lab.

### Determination of gene expression values using quantitative high-throughput RT-qPCR analysis

The mRNA was isolated from the frozen liver tissue samples using the Qiagen RNeasy Mini Kit (Valencia, CA). The mRNA quantity was measured using Nanodrop (Thermo Scientific, Waltham, MA). The RNA integrity number (RIN) was checked using Agilent 2100 Bioanalyzer (Agilent Technologies, Santa Clara, CA) and was above 8.5 for all samples. cDNA synthesis was performed with 2µL of 50 ng/µL total RNA, 1µL of 10 × TaqMan RT Buffer, 2.2µL 25 mM MgCl2, 2µL of 2.5 nM dNTP-Mix, 0.5µL of 50 µM random hexamers, 0.2µL of RNase Inhibitor, 0.25µL of 50 U/µL Multiscribe reverse transcriptase, and 1.85µL RNase-free water. All reagents were purchased from Applied Biosystems (TaqMan Reverse Transcription Reagents: N808-0234). The reaction mixtures were mixed with the RNA and incubated at 25 ℃ for 10 min, at 48 ℃ for 30 min, and then at 95 ℃ for 5 min. The generated cDNAs were run on a 96.96 microfluidic Dynamic Array™ IFC (Fluidigm Corporation, CA, USA), using a BioMark Instrument 76 (GE96X96 Standard v1.pcl – protocol file) and analyzed with Real-Time PCR 182 Analysis Software in the BIOMARK instrument (Fluidigm Corporation, CA, USA). From the cDNA samples, 3 technical replicates were used for the amplification step. Gene-specific primers were purchased from Life Technologies (Darmstadt, Germany). A listing of all primers is provided in the Additional file 1: (S1).

### Normalization of gene expression values using the linear (L) error (E) model (M)—ming method ( LEMming)

The analysis of qPCR raw data was performed using the LEMming method for the Fluidigm platform [30]. The Fluidigm platform with its parallel qPCR measurements implicates an experimental design that allows the estimation and exclusion of technical errors and thus a data normalization independent from reference genes. Thus, normalization using LEMming is based on a linear model including a number of effect variables that are estimated in the following order:

- 1. Probe error per array (*ε*_P:A_).
- 2. Systematic batch effects ($\stackrel{`}{\varepsilon }$).
- 3. Treatment/tissue effect (Δ_T_).
- 4. Sample error (ε_S_).
- 5. Treatment effect per gene (Δ_T:G_).

According to the linear model shown in Eq. 1, each measurement Y of a gene is a composition of these effects.

1 $$Y \, = \varepsilon_{P:A} + \varepsilon_{S} + \Delta_{T} + \Delta_{T:G} + \varepsilon$$

The variable *ε* is called residual and describes biological variance and non-systematic technical errors. The sum of Δ_T_ + Δ_T:G_ is used to calculate the fold change in comparison to a control condition.

Pre-processed LEMming cycle threshold (CT)-values were then transformed into 2^ΔCT^ expression values and normalized to the untreated control samples. The normalization using LEMming and further computational analyzes were performed using R [31]. The list of all gene expression data is included in the Additional file 2: (S2).

### Clustering of sample groups based on gene expression data

**Fig. 2:**
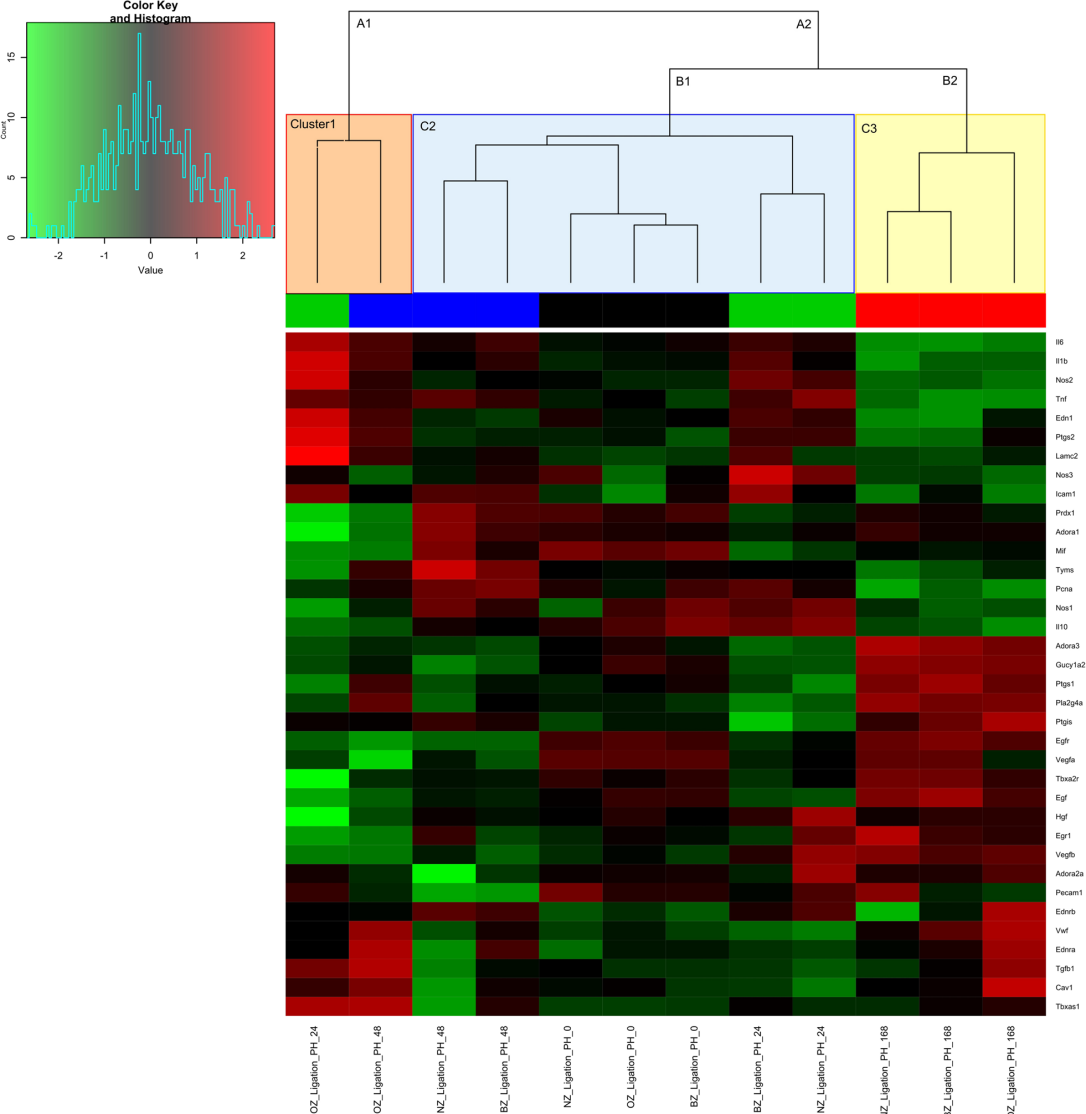
Heat-map with dendrogram. Heat map showing two-way hierarchical clustering of expression levels of 37 genes (rows) in each sample group (columns). Red cells indicate high expression, green low, black intermediate. There are four groups of samples—black sample labels represent 0 h post-OP, green labels represent 24 h post-OP, blue labels represent 48 h post-OP and red labels represent 168 h post-OP. The dendrogram shows three clusters of sample groups (C1-red, C2—blue, and C3-yellow)

The time*-*dependent expression of genes across tissue zones was analyzed using hierarchical clustering and principal component analysis (PCA). The hierarchical clustering was used to group samples according to gene expression profiles over time and all three zones (NZ, BZ, and OZ). The gene expression values were graphically represented in the heatmap. The samples were sorted according to the correlations of their gene expression profiles and their clustering presented in a dendrogram. The hierarchical clustering was visualized using the “gplots” package for R [32] (Fig. 2).

**Fig. 3:**
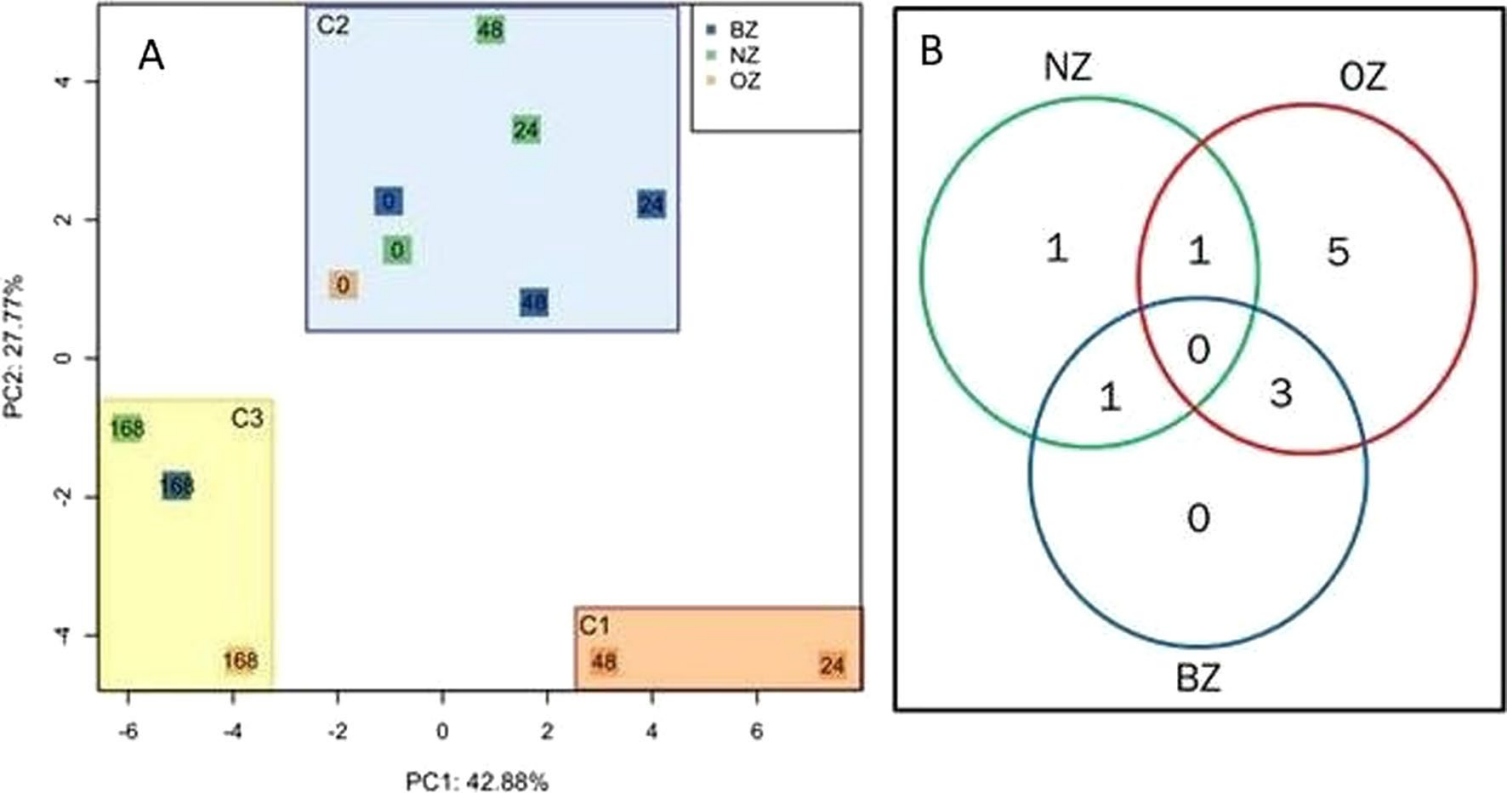
Clustering by gene expression: **A** Principle component analysis (PCA). Principle component analysis (PC1 versus PC2) based on transcriptome data of 37 genes in 12 sample groups (NZ- normal Zone (green); OZ—obstruction Zone (red); BZ- border Zone (blue)) and the observation time (0 h, 24 h, 48 h, and 168 h post-OP). The sample groups could be grouped into three clusters (C1-red, C2-blue, and C3-yellow). **B** Venn diagram. Venn diagram grouped 11 DEGs (adjusted *p*-value < 0,05 and fold change ≤ 0,33 or ≥ 3) within the three tissue zones (NZ, BZ, and OZ). Numbers represent the numbers of genes detected in tissue zones or their various overlapping subsets

Additionally, PCA was used to visually group samples based on gene expression profiles in a scatter plot. PCA is a multivariate technique that reduces the high dimensionality of the data. A multivariate dataset is visualized as a set of coordinates in high-dimensional data space (1 axis per gene corresponding to 37 dimensions in this study). Using this method, a smaller set of coordinates was determined in a retransformed multi-dimensional space. The reduction of coordinates was done by determining a new eigenvector that captures maximum variance in the original high-dimensional data. Based on the reduction of dimensions and the visualization of data with the highest variance, patterns within the data were recognized [33] (Fig. 3A).

### Identification and clustering of differentially expressed genes (DEGs) using linear models for microarray data (LIMMA)

LIMMA was used for the identification of DEGs [34]. Identification of DEGs over time (0, 24, 48, and 168 h post-op) compared to control (untreated) was performed for each of the tissue zones (NZ, BZ, and OZ) independently. Differential expression was assessed based on a Benjamini–Hochberg corrected *p*-value < 0.05 in combination with a threefold change in expression. The list of all gene expression data is included in the Additional file 2: (S2).

### Clustering of DEGs

The zonal distribution of DEGs was investigated using a Venn diagram. The Venn diagram visualized the numeric distribution of DEGs within the three tissue zones (NZ, BZ, and OZ).

The three tissue zones were visualized using circles. The numbers indicated in the circles correspond to the number of DEGs in the respective tissue zones. The number of DEGs that were differentially expressed in more than one tissue zone was indicated in the overlapping circles (Fig. 3B).

## Results

### Bioinformatic analysis

#### Clustering of data using hierarchical clustering and PCA

Two independent clustering algorithms revealed the same grouping of samples.

Hierarchical clustering with dendrogram grouped the samples based on the similarity of their gene expression profile (Fig. 2). As shown in the dendrogram, the analysis resulted in two main clusters (A1 and A2). The separation of the samples in the main clusters A1 and A2 visualized the strong differences in gene expression profiles between OZ samples at 24 h and 48 h (C1) and all other groups (A2). In contrast to A1 containing the only C1, cluster A2 was further divided into 2 large sub-clusters. Subcluster B1 contained all remaining samples, except the samples obtained at the end of the observation period of 168 h (C2), which formed a separate cluster (B2—C3).

By performing PCA, we found the first two principal components (PCs) to account for 70, 7% of the original biological variability in the dataset (PC1: 42,88%, PC2: 27,77%) (Fig. 3A) and three clearly separated clusters corresponding to the dendrogram. Cluster 1 (C1) enclosed the 24 h and 48 h samples from the OZ, C2 contained all 0 h samples as well as 24 h and 48 h samples from NZ and BZ and last but not least, C3 included all 168 h samples.

Hierarchical clustering and PCA showed that the greatest differences in gene expression occur at the time points 24 h and 48 h. For this reason, we concentrated on these two time points with the greatest changes for further evaluation.

#### Visualization of data using a venn diagram

Using the LIMMA analysis, we identified significantly DEGs (adjusted *p*-value < 0,05 and fold change ≤ 0,33 or ≥ 3) at 24 h and 48 h postoperatively compared to control (untreated). Using this cutoff, we identified 11 DEGs (Table 2). Complete results are shown in Additional file 2: data (S2).

**Table 2:**
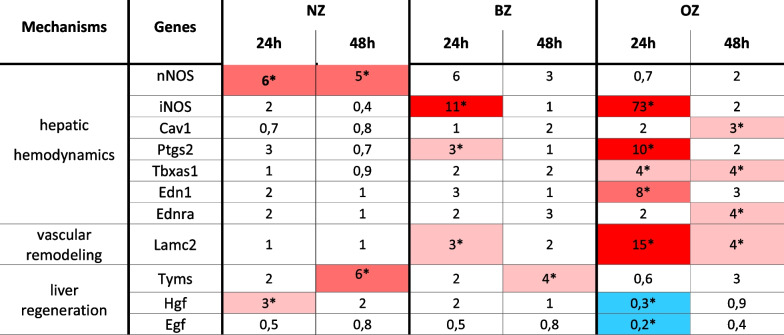
Differentially expressed genes (expressed as fold-change) compared to the control group

^*^adjusted *p*-value < 0,05; color code to highlight fold change ≤ 0,33 (blue) or ≥ 3 (light red), ≥ 5 (red), ≥ 10 (bright red), compared to control (untreated)

We visualized the number of differentially expressed marker genes according to the distribution in the different tissue zones in a Venn diagram (Fig. 3B). However, we observed a striking imbalance between the numbers of DEGs in OZ compared to the other two zones. The diagram depicts that five DEGs were found only in the OZ, whereas in the NZ and BZ only one gene and no gene was differentially expressed respectively. This pattern of distribution correlates well with the patterns revealed by the dendrogram and PCA, in which the OZ also differed significantly from the other zones.

#### Analysis of DEGs in respect to the three perfusion zones (OZ, BZ, NZ) in the liver

##### OZ

The gene analysis showed that 7 genes were upregulated, and 2 genes were downregulated in OZ at 24 h and 48 h post-OP compared to control (untreated). Among the DEGs, iNOS (73-fold), Lamc2 (15-fold), Ptgs2 (tenfold), and Edn1 (eightfold) emerged as the most upregulated genes at 24 h postoperatively (Table 2). These genes are involved in three hepatic hemodynamic regulatory mechanisms: NO pathway (iNOS), arachidonic acid pathway (Ptgs2), and endothelin pathway (Edn1). These upregulated genes are also known to be involved in proinflammatory responses [42, 43]. In particular, iNOS was strikingly more upregulated (70-fold) than the marker genes of other hepatic hemodynamic pathways. This finding suggests that the NO pathway could potentially be targeted for the reduction of damage from outflow obstruction after extended liver resection.

Another highly upregulated gene in OZ was Lamc2 (15-fold), a marker gene for vascular remodeling and a component of the basal lamina.

The following genes: iNOS (11-fold), Ptgs2 (threefold), and Lamc2 (threefold) were also over-expressed in BZ (Table 2).

In OZ, the most downregulated genes were EGF (0,twofold) and HGF (0,threefold) (Table 2). These genes encode growth factors relevant for liver regeneration. Downregulation of gene expression corresponded to the inhibition of proliferation observed in histology at this observation time point [26].

##### NZ

In contrast, our analysis revealed that only 3 genes, nNOS, Tyms and HGF, were moderately upregulated in NZ at 24 h and 48 h post-OP (3- to sixfold) compared to control (untreated) (Table 2). Overexpression of nNOS suggests an effect on vasodilatation which corresponded to the obvious sinusoidal dilatation occurring in the NZ at this time point. Overexpression of HGF and Tyms did fit well to the regenerative response of hepatocytes in the NZ. The cell cycle-associated marker Tyms was also moderately overexpressed (fourfold) in the BZ, also corresponding to the observed proliferative response (Table 2).

## Discussion

### Justification of selective gene expression profiling

In this study, we used selective gene expression profiling analysis to investigate a complex biological process. Quantitative high-throughput RT-qPCR by 96.96 microfluidic Dynamic Array™ from Fluidigm enables the investigation of many samples. In our study, we examined 6 rats (biological replicates) at each observation time point 0 h, 24 h, 48 h, and 7days. We analyzed three perfused regions (NZ, BZ, OZ) per rat liver. We applied 3 technical replicates of all samples to the "Dynamic Array™ IFC" Fluidigm chip. This resulted in a total number of 216 samples. Hence, differential gene expression analysis which is used for whole-genome gene expression profiling is far too expensive for that many samples. Based on well-known signaling mechanisms interwoven in hepatic hemodynamics, vascular remodeling, and liver regeneration we focused our analysis on 37 genes with specific interest for this scientific question.

### Identification of molecular pathways underlying the histological findings

One of the factors decisive for the outcome is the prevention of arterial hypoperfusion and subsequently the prevention of hepatic necrosis. Since, as reported before, all previously selected drugs: molsidomine, isosorbide-5-mononitrate, sildenafil, carvedilol, terlipressin, and octreotide, did not affect hepatic hemodynamics, we wanted to identify key regulatory mechanisms using selective gene profiling.

First, we confirmed the suitability of our strategy. Therefore, we first compared the expression of selected marker genes with the expected corresponding histological findings in terms of liver regeneration and vascular remodeling. Second, we analyzed the results of the expression level of the marker genes governing hepatic hemodynamics.

The bioinformatic analysis (hierarchical clustering and PCA) revealed that differences in gene expression were most prominent at 24 h and 48 h and occurred predominantly in the OZ (Venn diagram) compared to the other time points and zones (Fig. 2, 3). These findings corresponded to the previous histological observations showing that the most obvious changes occurred indeed in the OZ at this time point [26].

Gene expression analysis showed that the liver regeneration marker (Tyms) was the most upregulated gene in NZ and BZ, corresponding to the pronounced hepatocyte proliferation. In contrast, other markers of liver regeneration (HGF and EGF) were markedly downregulated in OZ at 24 h corresponding to the absence of hepatocyte proliferation in this region. Similarly, we observed that upregulation of the vascular remodeling marker Lamc2 corresponded well with the formation of vascular canals in OZ and BZ of these animals as previously described by Huang et al. (2014) [26]. These findings suggest that the selected marker genes were indeed indicative of the histological alterations.

Regulation of hepatic hemodynamics is based on four intermingled key signaling pathways: Adenosine-based HABR, NO pathway, arachidonic acid pathway, and endothelin pathway.

#### Adenosine-based HABR

HABR leads to vasodilatation via the regulation of the adenosine concentration in the "Space of Mall". The adenosine in the "Space of Mall" binds to the adenosine receptors of the hepatic arterial vessel wall. If the adenosine receptors are activated, arterial vascular dilatation occurs.

We investigated the expression of adenosine receptor genes: Adora1, Adora2a, and Adora3 (Fig. 1).

All three adenosine receptor genes were not differentially expressed at 24 h or 48 h after the operation. This result corresponds to the observations of the studies by Dold et al. (2015) and Audebert et al. (2017) [44, 45]. Dold et al. (2015) observed that portal hyperperfusion after 70% and 90% hepatectomy did not induce a HABR [44]. Audebert et al. (2017) investigated hemodynamic changes during partial liver resection and created a computational model of hepatic hemodynamics. They observed a 75% decrease in hepatic arterial blood flow during surgery [45]. Based on their simulation, they showed that this 75% decrease in hepatic arterial flow can be explained by the increase in resistance induced by the surgical procedure itself.

These and our studies suggest that the HABR is not necessarily required for the regulation of liver perfusion in this situation. Thus, targeting the HABR does not seem to be a suitable strategy to influence liver perfusion after extended liver resection.

#### NO pathway

NO pathway leads to vasodilatation via conversion of L-arginine by activation of guanylate cyclase (Gucy1a2) (Fig. 1). NOS catalyzes the production of NO from L-arginine. eNOS, iNOS, and nNOS are three isoforms of NOS. NO increases the activity of guanylate cyclase, thereby increasing the concentration of cyclic guanosine monophosphate (cGMP), which in return leads to vasodilatation.

We selected 4 genes as marker genes from the NO pathway: eNOS, iNOS, nNOS, and Gucy1a2 (Fig. 1).

Both eNOS and Gucy1a2 were not differentially expressed, neither at 24 h nor at 48 h after the operation.

**Fig. 4:**
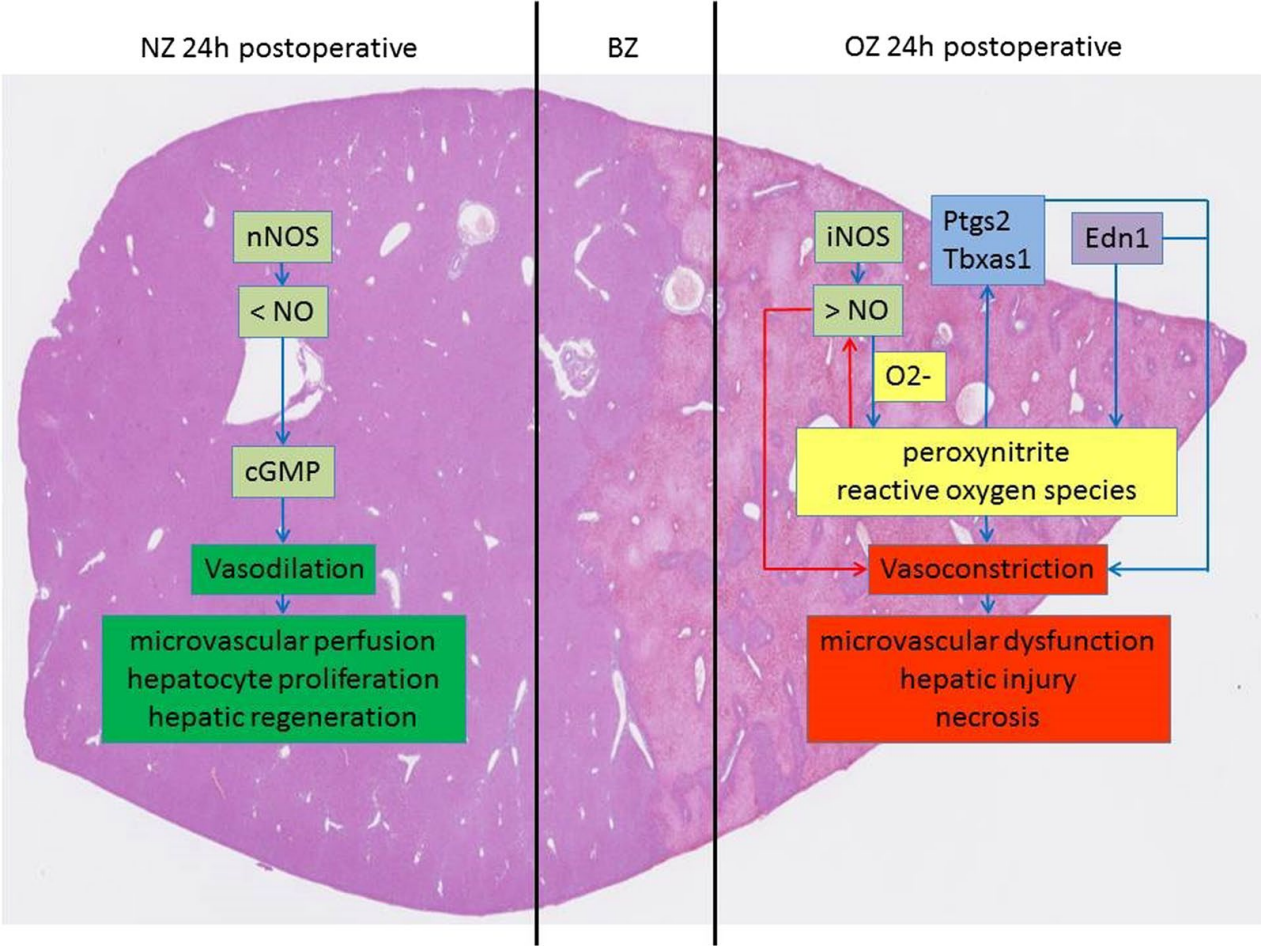
Regulation of hepatic hemodynamics in NZ and OZ. Diagram of the possible interactions regulating hepatic hemodynamics in NZ and OZ based on our results and the literature data. (light green box—NO pathway, blue box—genes of the arachidonic acid pathway, violet box—genes of the endothelin pathway, yellow box—formation of oxygen species, dark green box—effect of vasodilatation, red box—effect of vasoconstriction, blue arrows—enhancing effect, red arrows—inhibitory effect)

In contrast, nNOS mRNA-expression increased by sixfold, but only in NZ. nNOS is constitutively expressed and leads to the generation of only small amounts of NO [46]. nNOS increases vascular cGMP production and promotes vasodilatation [47] (Fig. 4). However, NO is a double-edged sword. Low levels of NO lead to vasodilation such as the NO release by nNOS [48]. In contrast, high levels of NO promote vasoconstriction, which might contribute to microvascular dysfunction and hepatic injury [49, 50] (Fig. 4). iNOS is the inducible isoform of NOS and generates 1000-fold larger quantities of NO than nNOS [27]. In our study, iNOS was the highest up-regulated gene (73-fold) in OZ. One of the reasons for the microvascular dysfunction and hepatic injury after upregulation of iNOS might be due to the reaction of NO with O_2_^−^ to form cytotoxic peroxynitrite and other reactive oxygen species [27, 51]. Peroxynitrite reduces the NO bioavailability for vasodilation [52, 53]. Furthermore, peroxynitrite can modify cellular macromolecules and may aggravate adenosine triphosphate depletion, leading to hepatocyte and endothelial cell necrosis [54, 55] (Fig. 4). McNaughton et al. (2002) showed that in human cirrhotic livers, there was a significant increase in iNOS in the cirrhotic areas [56]. Li and Billiar (1999) reported that suppression of iNOS could represent a therapeutic strategy to prevent liver damage, as upregulation of iNOS expression appears to involve the coproduction of reactive oxygen species [57]. Therefore, selective inhibition of iNOS could be a possible strategy to reduce vasoconstriction and the resulting tissue damage and formation of necrosis, as observed in the OZ.

This strategy may not only be promising for hepatic outflow obstruction, but also also in other liver diseases. Hazam et al. observed a significant positive correlation between iNOS and eNOS levels compared with the severity of disease parameters of HEV-related acute hepatitis in a study population. In addition, they found that high levels of iNOS and eNOS were associated with an increased risk of HEV-related acute hepatitis and liver failure [58]. Tache et al. showed that infection with hepatitis B and C viruses induces iNOS expression in hepatocytes, suggesting that NO overproduction might have an important role in progression of chronic viral hepatitis to cirrhosis [59]. Based on the evaluation of 168 publications, Iwakiri and Kim describe in their review the significance of nitric oxide in various clinical liver diseases as fatty liver disease, viral hepatitis and hepatic fibrosis [60]. Thus, the investigation of iNOS seems to be important not only for our model, but also for various other clinical liver diseases.

#### Arachidonic acid pathway

Activation of the arachidonic acid pathway can cause either vasodilatation or vasoconstriction. Upon activation of the arachidonic acid pathway, arachidonic acid is released from the cell membrane by the enzyme phospholipase A2 (Pla2g4a). The free arachidonic acid undergoes oxidation by Ptgs1 or Ptgs2 to prostaglandin G_2_ and further to prostaglandin H2. In endothelial cells, prostaglandin H2 is converted into prostacyclin by Ptgis and acts as a vasodilator. In contrast, prostaglandin H2 is metabolized into thromboxane by Tbxas1 in Kupffer cells. Thromboxane binds to Tbxa2r and acts as a vasoconstrictor.

We selected 6 genes as marker genes: Pla2g4a, Ptgs1, Ptgs2, Ptgis, Tbxas1, and Tbxa2r (Fig. 1).

In this study, the genes associated with vasoconstriction such as Ptgs2 and Tbxas1 were upregulated in BZ and OZ at 24 h and 48 h post-op. Ptgs2 was the second highest upregulated vasoactive gene in OZ at 24 h post-op. This upregulation of Ptgs2 mRNA expression corresponds to the observations published by Mohammed et al. (2004) and Schmedtje et al. (1997) [61, 62]. They also reported that the gene expression of Ptgs2 was induced after hepatic injury. Ptgs2 and thromboxane upregulation promote vasoconstriction in the presence of peroxynitrite, which is produced depending on NO-levels [62, 63] (Fig. 4).

Therefore, inhibition of iNOS could be an interesting strategy to reduce the detrimental vasoconstriction mediated by Ptgs2 and thromboxane.

#### Endothelin pathway

Activation of the endothelin pathway can also result in both: vasodilatation and vasoconstriction. Endothelin is synthesized and released by smooth muscle cells, endothelial cells, and Ito cells. The isoforms ET-1, ET-2, and ET-3 can bind to the ET receptors type A or B (1/2). In the liver, ET causes vasoconstriction by binding to ET-A receptors on perisinusoidal Ito cells or by binding to ET-B2 receptors on endothelial cells and Kupffer cells. In contrast, the binding of ET to the ETB1 receptor stimulates the endothelium to produce and release prostacyclin. It also activates eNOS and causes vasodilatation by releasing NO [65–67].

We selected 3 genes as marker genes: Edn1, ET-R_A_, and ET-R_B_ (Fig. 1).

In this study, ET-R_A_ and Edn1 promoting vasoconstriction were upregulated in OZ. Edn1 was the third highest upregulated vasoactive gene in OZ. The upregulation of Edn1 corresponds to the findings of Earley et al. (2002), who observed that NO also causes an increased release of Edn1. Other studies have shown that Edn1 leads to a decrease in sinusoidal volumetric flow by vasoconstriction [68–70]. In addition, Edn1 increases the production of peroxynitrite [71] (Fig. 4).

Altogether, this suggests that inhibition of iNOS-expression could also lead to a reduction of vasoconstriction by Edn1.

### Druggable signal transduction pathways

Overproduction of vasoconstrictors and impairment of vasodilatation may lead to an imbalance in hepatic hemodynamics. The study of Liang et al. (2003) reported that microcirculatory injury in small-for-size liver grafts resulted in upregulation of mRNA expression of Edn1 (2.5- to sixfold) and iNOS (6.4- to 24-fold) [72]. Furthermore, they showed that the upregulation of Edn1 and iNOS leads to a deterioration of intracellular homeostasis.

Imbalance in hepatic hemodynamics may promote hepatic damage. The imbalances between vasoconstrictors (mainly induced by upregulation of Edn1 and cyclooxygenase-derived prostaglandins) and impaired vasodilation (mainly NO) are responsible for the increased vascular tone in the sinusoidal and postsinusoidal space, vasoconstriction and narrowing of the sinusoidal lumen, compromising blood flow, tissue oxygenation, and cell trafficking. Also, these imbalances together with the increased intrahepatic resistance are important for the pathophysiology of portal hypertension in cirrhotic livers [23, 73].

As mentioned before, modulation of NO pathway by the non-specific NOS-inhibitor L-NAME impaired hepatic microcirculation and aggravated parenchymal damage after extended liver resection [26]. L-NAME leads to feedback regulation of NO expression and an increase of iNOS. The feedback regulation is followed by inhibition of eNOS and nNOS mediated by L-NAME. By inhibiting the eNOS and nNOS, the NO level may decrease. The decrease in NO level leads to transcription factor nuclear factor kB (NF-kB) activation. NF-kB, a key factor in iNOS expression, increased iNOS expression [55, 74, 75]. This could possibly explain that L-NAME impaired hepatic microcirculation and aggravated parenchymal damage after extensive liver resection. Therefore, modulation of iNOS should be investigated instead of using a non-specific modulation of the NO pathway as pursued before.

These considerations suggest in particular that the specific inhibition of iNOS expression could be a promising strategy to reduce liver damage after extensive liver resection. It appears that high NO levels due to the elevated iNOS expression lead to vasoregulatory imbalance and an increase of hepatic damage. This has already been investigated in other studies (Table 3). The administration of specific iNOS inhibitors such as Aminoguanidine, ONO-1714, Sivelestat, and 1400 W showed improvements in liver injury in experimental studies of ischemia–reperfusion and PHx [76–83]. Other strategies to modulate iNOS include expression control methods, e.g., using microRNAs or antisense RNA. There are interesting studies on the application of microRNAs or antisense RNA for instance in inflammatory livers or hepatocytes [84, 85]. In view of these publications, we speculate that modulation of the NO signaling pathway by specific inhibition of iNOS expression could be a possible signal transduction pathway to also reduce liver damage due to outflow obstruction after extended liver resection. In the next study, we will further investigate the effect of iNOS and the downregulation of iNOS in our model.

**Table 3:** Examples of studies on the regulation of iNOS in the liver

Drug	Treatment	Species	Result	References
Aminoguanidine	Phx	rat	Decreased iNOS protein level	[75]
	LPS	rat	Attenuated LPS-induced hepatotoxicity	[76]
ONO-1714	Phx	pig	Reduced liver damage	[77]
	I/R	pig	Reduced liver ischemia–reperfusion damage	[78]
	I/R	pig	Reduced liver damage & stabilized hemodynamics	[79]
Sivelestat	I/R	rat	Prevented hepatic I/R injury	[80]
1400 W	transplantation	rat	Effective therapy for primary nonfunction of fatty liver grafts	[81]
	I/R	rat	Decreased NO metabolites level in serum	[82]
	ICC	tissue / cell	Suppressed cell proliferation, invasion, and migration in ICC	[83]
microRNA149	LPS	mice	Reduced inflammation in liver	[84]
asRNA	–	HC rat	Reduced iNOS mRNA levels	[85]

*asRNAs* Antisense RNA; *HC *Hepatocytes cells, *I/R* Ischemia–reperfusion, *Phx* Partial hepatectomy, *ICC* Intrahepatic cholangiocarcinoma

## Conclusion

In our study, the NO pathway turned out to be the most affected pathway from the four investigated vasoactive pathways. Due to the central role of iNOS in the intermingled vasoactive pathways, selective downregulation of iNOS-expression seems to be the most promising approach to reduce the risk of post-operative liver failure.

## Supplementary Information

**Additional file 1: ** List of gene primers.

**Additional file 2:** List of all normalized gene expression data.

## Data Availability

All data generated or analysed during this study are included in this published article and its Additional information files.

## Abbreviations

BZ: Border zone
CT: Cycle threshold
cGMP: Cyclic guanosine monophosphate
DEGs: Differentially expressed genes
FHOO: Focal hepatic outflow obstruction
HABR: Hepatic arterial buffer response
LEMming: Linear error model—ming
LIMMA: Linear models for microarray data
L-NAME: N(*ω*)-nitro-L-arginine methyl ester
NO: Nitric oxide
NOS: NO synthase
NZ: Normal zone
OZ: Outflow obstruction zone
PCA: Principal component analysis
PHx: Partial hepatectomy
RIN: RNA integrity number
RT-qPCR: Quantitative reverse transcription PCR
RMHVL: Right median hepatic vein ligation
